# A case of spinal metastasis with osteoblastic lesion: The risk of epidural extension and paralysis

**DOI:** 10.1002/ccr3.9464

**Published:** 2024-10-09

**Authors:** Tasuku Ino, Yu Toda, Hirohito Hirata, Tomohito Yoshihara, Masatsugu Tsukamoto, Masaaki Mawatari, Tadatsugu Morimoto

**Affiliations:** ^1^ Department of Orthopaedic Surgery, Faculty of Medicine Saga University Saga Japan

**Keywords:** colorectal carcinoma, malignant spinal cord compression, metastatic lesion, osteoblastic type

## Abstract

One patient with metastatic spinal tumors from colorectal cancer experienced malignant spinal cord compression (MSCC). Determining the extent of the lesion using magnetic resonance imaging is essential, even if the lesion is osteoblastic. Therefore, it is important to evaluate the risk of MSCC in patients with metastatic spinal tumors.

## 1 INTRODUCTION

Due to an aging society and improvements in anticancer therapy, patients with cancer can survive with bone metastasis. Bone metastasis decreases a patient's quality of life (QOL) and activities of daily life (ADLs) due to skeletal‐related events (SREs), such as pain and pathological fractures and malignant spinal cord compression (MSCC). In recent years, we have emphasized the importance of managing spinal metastasis, including treatment.^1^ Among SREs, MSCC reduces the QOL and ADL of patients with cancer and shortens life expectancy. Past literature showed that MSCC incidence occurs in 2.5%–5% of patients with malignant neoplasm.^2^ As a rule, MSCC occurs in patients who already have metastatic spinal tumors.^3^ However, MSCC sometimes would be the trigger for the first diagnosis of malignant neoplasm, most commonly in patients with lung cancer.^4^ Radiologically, bone metastasis is identified, followed by classification of the osteolytic, osteoblastic, mixed, and intertrabecular subtypes. Among the radiological types of bone metastases, the risk of spinal cord compression varies. Compared with the osteolytic type, the osteoblastic type scores are lower in the Spinal Instability Neoplastic Score (SINS), which determines the stability of the spine with a metastatic spinal tumor. The osteolytic type can also radiologically change to an osteoblastic lesion after radiotherapy or chemotherapy; the osteoblastic type tends to be neglected in subsequent magnetic resonance imaging (MRI) or other imaging evaluations and is considered a treatment response case. Therefore, osteoblastic lesions are often neglected as potential therapeutic targets. However, it should be noted that tumor extradural extension may occur and cause paralysis, even if it is thought to be the sclerotic type. Herein, we report a case of a metastatic spinal tumor that showed an osteoblastic type that unfortunately caused paralysis.

## 2 CASE PRESENTATION

### 2.1 Case history and examination

**FIGURE 1:**
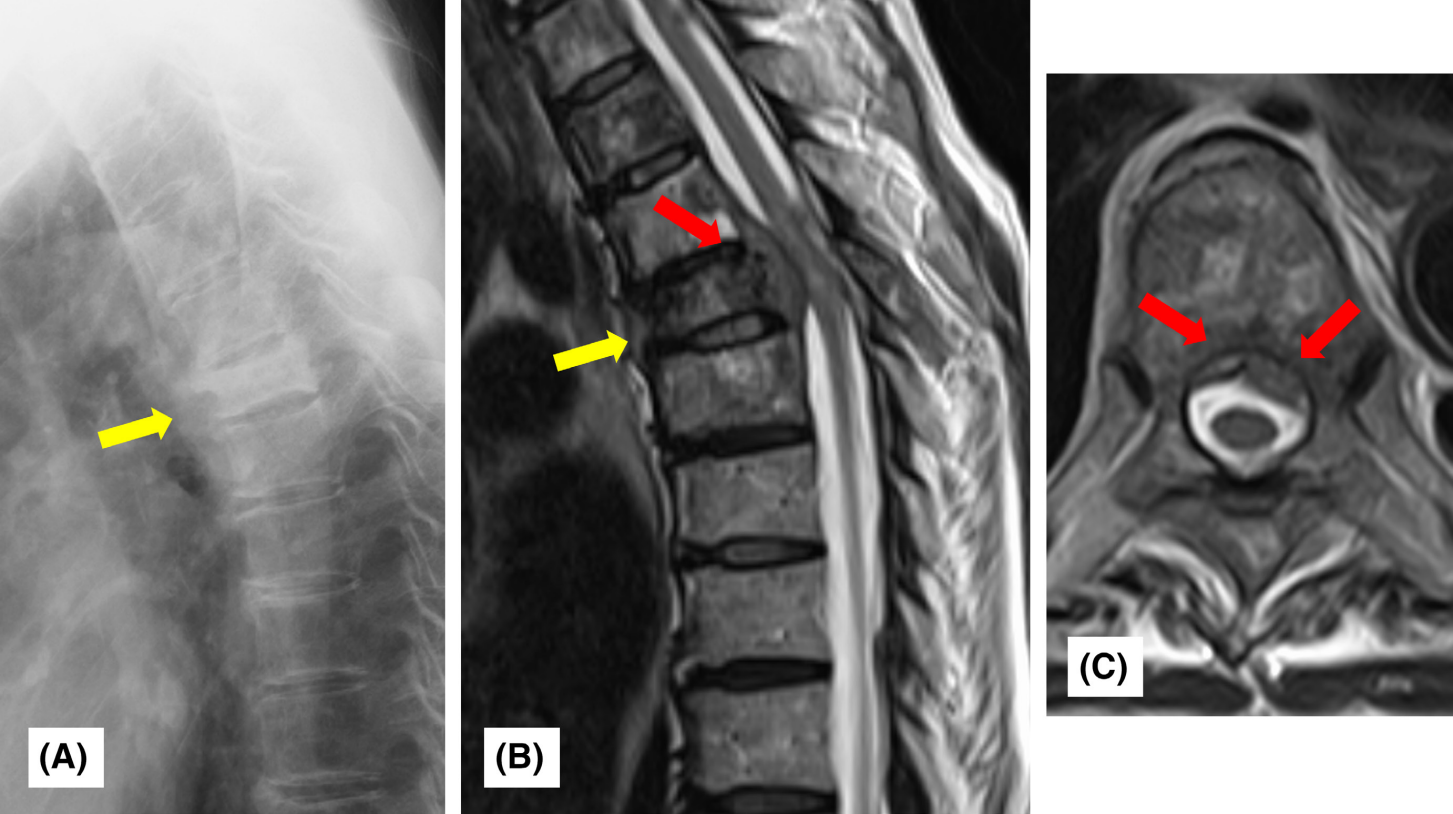
Preoperative image findings. (A) Plain radiography showed the osteoblastic lesion of vertebra T7 with decreased height (yellow arrow). (B) Plain spinal magnetic resonance imaging on sagittal T2 weighted imaging (WI) showed the intravertebral tumor (yellow arrow) and extraspinal tumor compressing the spinal canal (red arrow). (C) The mass (red arrows) bilaterally compressed the spinal canal, as demonstrated by axial T2WI.

Herein, we present the case of a 57‐year‐old woman with the rapid progression of bone metastasis from colorectal cancer. She underwent right hemicolectomy for colorectal cancer and was treated with TS‐1 + CPT11 as chemotherapy for 4 years after operation for the primary tumor. Eight years after the initial surgery, a recurrent tumor was detected in the right renal and periaortic lymph nodes. The patient was administered chemotherapy (XELOX + bevacizumab). One year after the change in the chemotherapy regimen, distant metastasis was observed at T7 of the spine. Therefore, zoledronic acid injections were initiated. Periodic radiological investigations considering prophylactic spinal fusion surgery were performed carefully. The patient remained pain‐free, and plain radiography and computed tomography (CT) revealed a change in the osteoblastic lesion (Figure 1A). We determined that her disease responded well to treatment. Therefore, we continued the follow‐up with additional treatment modalities. However, 2 years after changing her chemotherapy regimen, she rapidly developed paralysis in both lower extremities. MRI revealed that the tumor had developed into the spinal canal and compressed the spinal cord (Figure 1B,C). The patient was referred to our hospital for further surgery.

Upon admission to our hospital, the patient had weakness in both lower extremities with right predominance and bladder and rectal disturbances. The manual muscle test (MMT) score of the right lower extremity was almost 1–2 points, and that of the left lower extremity was almost 3. The patient had no upper extremity symptoms. Thermal hypoalgesia in the lower extremities was also observed, according to the Frankel C1 classification.

### 2.2 Treatment

**FIGURE 2:**
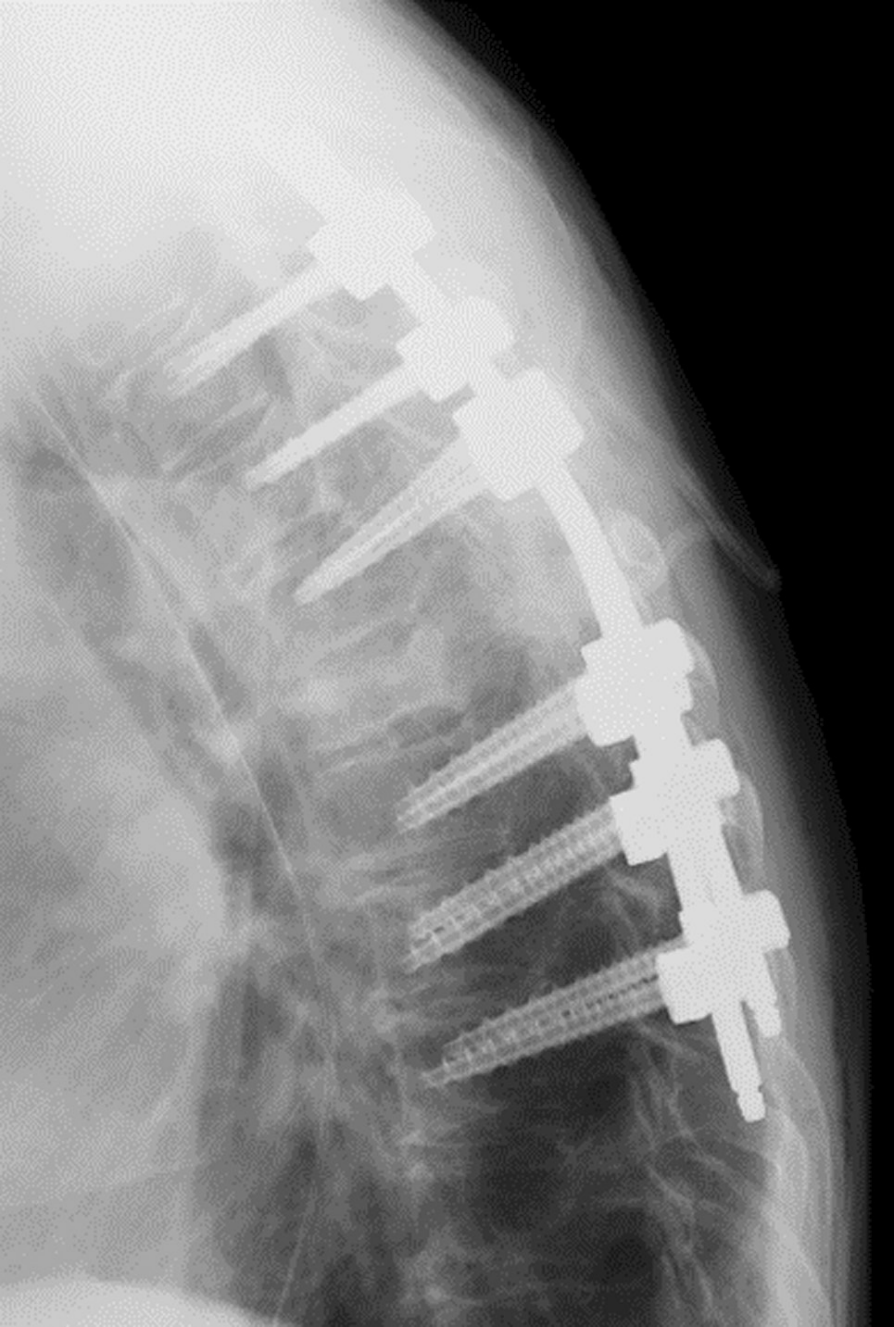
Postoperative image findings on plain radiography.

The patient was diagnosed with a metastatic thoracic spinal tumor caused by colorectal cancer. Emergency surgery was performed because of bladder and rectal disturbances and the rapid onset of paralysis. Laminectomy and posterior fusion of T3‐9 were performed (Figure 2).

### 2.3 Outcome and follow‐up

Postoperatively, the MMTs of the right lower extremity recovered to almost 2–3 points. Using a 4‐point brace, the patient held a standing position independently, albeit briefly. The patient was transferred to a hospital for rehabilitation 1 month after surgery and was scheduled to receive postoperative radiation therapy and chemotherapy with a modified regimen. However, after transfer to another hospital, lung metastasis developed, and her general condition gradually deteriorated. Postoperative rehabilitation did not progress well, and the final walking condition was ambulation on a parallel bar. Four years after the spinal surgery, the patient died.

## 3 DISCUSSION

### 3.1 Bone metastasis in colorectal cancer

Santini et al. performed the largest multicenter study, including 264 patients with bone metastasis from colorectal cancer, and showed the clinicopathological characteristics of patients.^5^ In their study, they found that the common anatomical site of bone metastasis from colorectal cancer was the spine (65%). Moreover, they investigated the radiographic characteristics of bone metastases from colorectal cancer. Interestingly, osteolytic lesions are common (80%) among bone metastases from colorectal cancer. However, osteoblastic lesions account for less than 8% of cases.^6^

### 3.2 Clinical features and diagnosis of MSCC

Previous literature discussed the route by which the tumor reached the spinal cord. One way is the growth of a paravertebral tumor into the spinal canal, passing through the intervertebral foramen.^3^ However, Algra et al. reported this was not a major route. In more than 85% of cases, the tumor reaches the spinal cord through an indirect route. It first spreads through the bloodstream to the vertebral body, where it grows within the bone. The tumor would extend into the epidural space and cause MSCC.^8^ Patients with MSCC are most initially aware of back pain, which appears as an initial symptom in 83 to 95% of patients,^9^, ^10^ followed by lower extremity weakness.^3^ In our case, there was no back pain, but lower extremity muscle strength was observed, which triggered the diagnosis of MSCC. MRI with high sensitivity (93%) and specificity (97%) is the most useful modality for diagnosing MSCC.^11^, ^12^

### 3.3 Management of osteoblastic spinal metastasis

Based on the spinal instability neoplastic score,^7^ the risk for spinal instability of osteoblastic lesions is lower than that of osteolytic lesions. Moreover, Pennington et al. analyzed the Hounsfield units (HUs) of metastatic spinal tumors on CT.^13^ They reported a significant positive correlation between lower HUs and pathological fracture.^13^ In the existing literature, cases with expanded bony elements of metastatic spinal tumors showed spinal cord compression.^14^, ^15^ However, among these cases, the metastatic spinal tumor directly compressed the spinal canal, and the pathophysiology differed from our case in which the tumor extended into the soft tissue. Osteoblastic lesions tend to require less surgical intervention than osteolytic lesions. To our knowledge, there are no previous reports on MSCC in osteoblasts. Some reports have examined the accuracy of the diagnosis of MSCC using CT,^16^, ^17^ but there is no mention of an association between the osteoblastic type and MSCC.

Patchell et al. conducted a clinical trial comparing only radiation and surgery with postoperative radiation among patients with spinal metastases and reported improvement in functional and life prognosis with the latter.^18^ However, decompression surgery may result in intertumoral resection, and oncological control is not possible. Some studies have reported the effectiveness of postoperative radiation therapy^19^ such as stereotactic radiosurgery^20^ and separation surgery,^21^ which contributed to improved local control and overall survival. However, our patient did not receive postoperative treatment because of disease progression.

### 3.4 Reflections on this case

**FIGURE 3:**
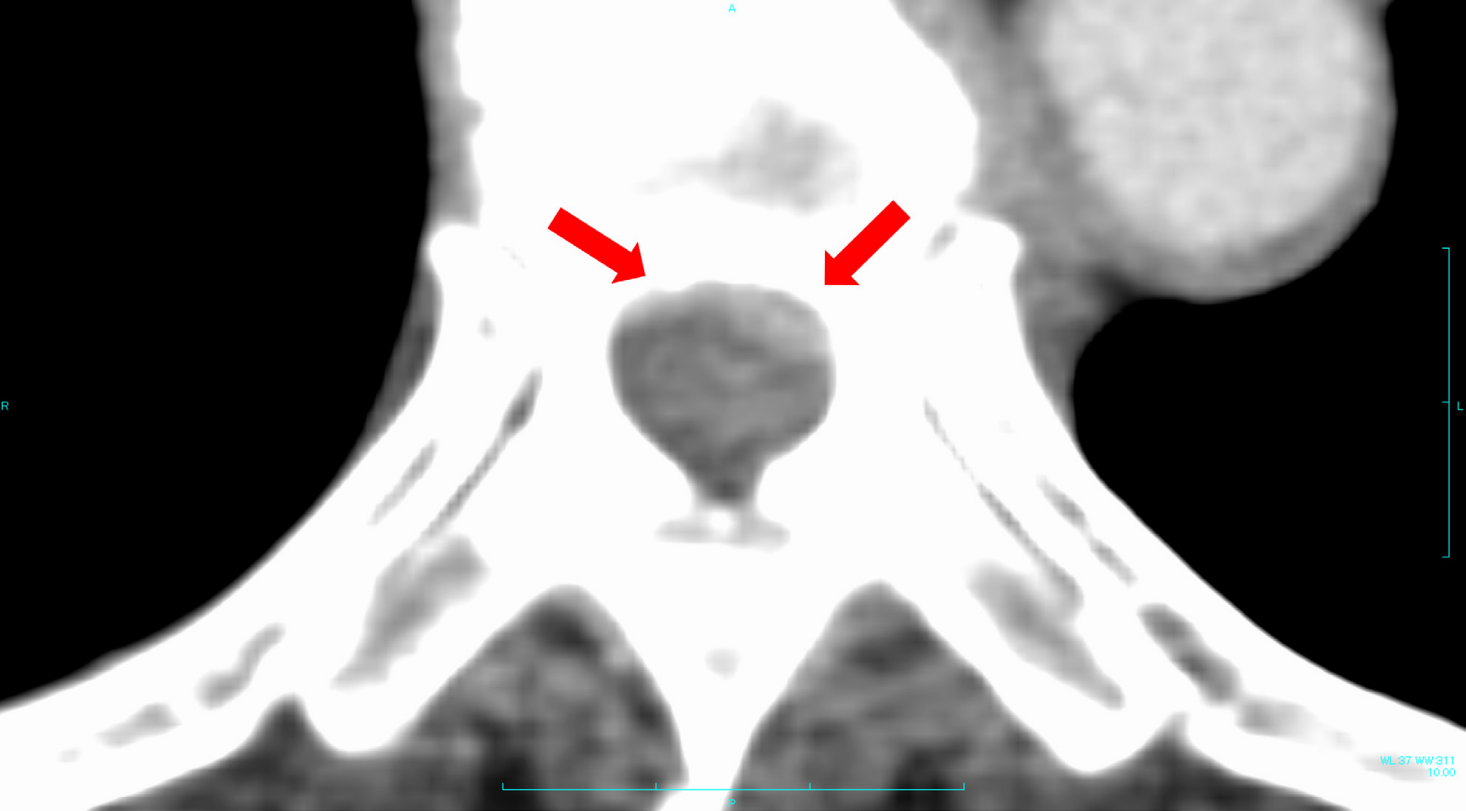
Contrast‐enhanced computed tomography detected an enhanced tumor (red arrows) invading the surrounding soft tissue, revealing malignant spinal cord compression. There is no evidence of bone destruction in these imaging findings.

Computed tomography revealed that the patient was osteosclerotic, responded to treatment, and underestimated the low risk of MSCC. To prevent metastasis, we should have investigated the soft tissue for epidural extension using CT. Moreover, periodic MRI is necessary even for osteoblastic lesions of metastatic spinal tumors. In retrospect, a contrast‐enhanced CT scan of the torso should have been obtained at a periodic follow‐up performed by the primary department before the onset of symptoms showed MSCC (Figure 3).

## 4 CONCLUSION

We encountered a case of rapid paralysis of the lower limbs due to MSCC caused by bone metastases from colorectal cancer. Osteoblastic spinal metastasis generally poses a lower risk of spinal instability; however, accurate imaging (i.e., MRI) and follow‐up evaluation are vital to assess potential complications such as MSCC.

## AUTHOR CONTRIBUTIONS

**Tasuku Ino:** Writing – original draft. **Yu Toda:** Writing – review and editing. **Hirohito Hirata:** Writing – review and editing. **Tomohito Yoshihara:** Writing – review and editing. **Masatsugu Tsukamoto:** Writing – review and editing. **Masaaki Mawatari:** Supervision; writing – review and editing. **Tadatsugu Morimoto:** Supervision; writing – review and editing.

## FUNDING INFORMATION

None.

## CONFLICT OF INTEREST STATEMENT

The authors declare no conflicts of interest.

## ETHICS STATEMENT

This study was performed in accordance with the Declaration of Helsinki. The patient was treated in accordance with clinical needs. Ethical review and approval were waived for this study because the treatments for this clinically treated patient were not originally intended to be the subject of a case report.

## CONSENT

Written informed consent was obtained from the patient to publish this report in accordance with the journal's patient consent policy.

## Data Availability

The data that support the findings of this study are available on request from the corresponding author. The data are not publicly available due to privacy or ethical restrictions.
